# Latent Factor Modeling Reveals Unexpected Spatial Heterogeneity in Human Alzheimer’s Disease Brain Transcriptomes

**DOI:** 10.34133/csbj.0108

**Published:** 2026-05-14

**Authors:** Rami Al-Ouran, Chaozhong Liu, Linhua Wang, Zhijian Yu, Ying-Wooi Wan, Chaohao Gu, Xiqi Li, Gerarda Cappuccio, Mirjana Maletic-Savatic, Aleksandar Milosavljevic, Joshua M. Shulman, Hu Chen, Zhandong Liu

**Affiliations:** ^1^ Department of Data Science and Artificial Intelligence, Al Hussein Technical University, Amman, Jordan.; ^2^ Jan and Dan Duncan Neurologic Research Institute, Texas Children’s Hospital, Houston, TX 77030, USA.; ^3^ Department of Pediatrics, Baylor College of Medicine, Houston, TX 77030, USA.; ^4^ Center for Drug Discovery, Baylor College of Medicine, Houston, TX 77030, USA.; ^5^ Department of Neuroscience, Baylor College of Medicine, Houston, TX 77030, USA.; ^6^ Department of Molecular and Human Genetics, Baylor College of Medicine, Houston, TX 77030, USA.; ^7^ Department of Neurology, Baylor College of Medicine, Houston, TX 77030, USA.; ^8^ Center for Alzheimer’s and Neurodegenerative Diseases, Baylor College of Medicine, Houston, TX 77030, USA.

## Abstract

Alzheimer’s disease is characterized by complex molecular and cellular heterogeneity, which complicates efforts to identify consistent biomarkers and therapeutic targets. To better characterize the heterogeneity, we applied latent factor modeling to RNA sequencing data from approximately 2,500 human Alzheimer’s disease brain samples, uncovering underlying patterns in gene expression. These transcriptional groups demonstrated unique gene expression profiles related to synaptic and neuronal pathways, vasculature development, and protein folding and antigen processing. Notably, this latent factor reflects variation in spatial sampling. Adjusting for the latent factor improved the identification of differentially expressed genes in disease samples. This finding suggests that spatial heterogeneity is a pervasive driver of transcriptomic variation and has important implications for future studies of Alzheimer’s disease and related neurological disorders.

## Graphical Abstract

**Figure:**
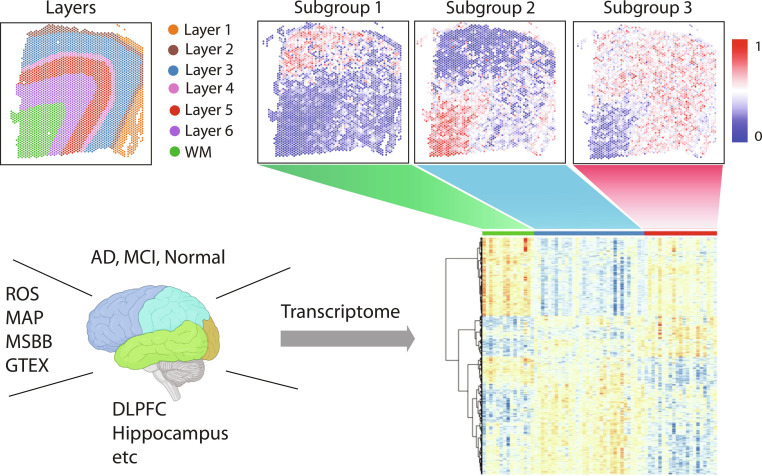

## Introduction

Alzheimer’s disease (AD), a progressive neurodegenerative disorder, currently affects ~6.7 million Americans aged 65 and older, a figure expected to rise to 13.8 million by 2060 [1]. The pathological hallmarks of AD include the accumulation of amyloid-beta plaques, neurofibrillary tangles, synaptic dysfunction, and widespread neuronal loss, which collectively contribute to the cognitive decline and memory impairments characteristic of the disease.

AD exhibits substantial heterogeneity across multiple biological scales, including molecular, cellular, and systems-level processes. This heterogeneity has been characterized using diverse modalities, such as transcriptomic profiling, biofluids, and neuroimaging [2,3]. Clinically defined subtypes of AD, such as basal, limbic-predominant, and hippocampal-sparing variants, exhibit distinct patterns of pathology and progression [4]. These subtypes highlight the complexity of the disease, emphasizing the need to explore its underlying molecular mechanisms. In parallel, researchers have identified diverse molecular signatures—spanning genetic, transcriptomic, proteomic, and metabolomic profiles—that provide further insights into the heterogeneity of AD. As one of the most successful large-scale initiatives, Accelerating Medicines Partnership for Alzheimer’s disease (AMP-AD) have revealed tremendous molecular heterogeneity in AD [5–8]. For example, Neff *et al.* [8] identified 3 major molecular subtypes of AD using an integrative network approach on 1,543 AD transcriptomes. The 3 subtypes were distinguished by varying levels of tau-medicated neurodegeneration, amyloid-β neuroinflammation, immune functions, myelination, and other dysregulated pathways.

Despite these advances, the search for molecularly defined AD subtypes is often hindered by clinical and technical confounders, such as age, gender, and batch effects. These confounders can introduce systematic biases that obscure meaningful biological patterns or generate spurious correlations. Traditional approaches to AD subtyping typically rely on predefined variables, which often fail to account for hidden or latent factors—unmeasured variables inferred from the data—that may drive molecular heterogeneity.

Latent factor modeling has proven to be a powerful approach in addressing these challenges in high-dimensional data analysis. Techniques such as guided principal component analysis [9], surrogate variable analysis [10], and probabilistic factor models (e.g., PEER [11]) have been instrumental in identifying hidden sources of variation and refining the interpretation of transcriptomic data. However, few studies have specifically focused on using latent factor modeling to uncover hidden structures in AD datasets that drive molecular heterogeneity. To address this gap, we conducted a rigorous statistical inference using a method called Data-Adaptive Shrinkage and Clustering (DASC [12]) to identify and interpret latent structures underlying distinct AD subtypes.

## Results

### Identification of a pervasive latent factor in brain transcriptome cohorts

**Fig. 1.**
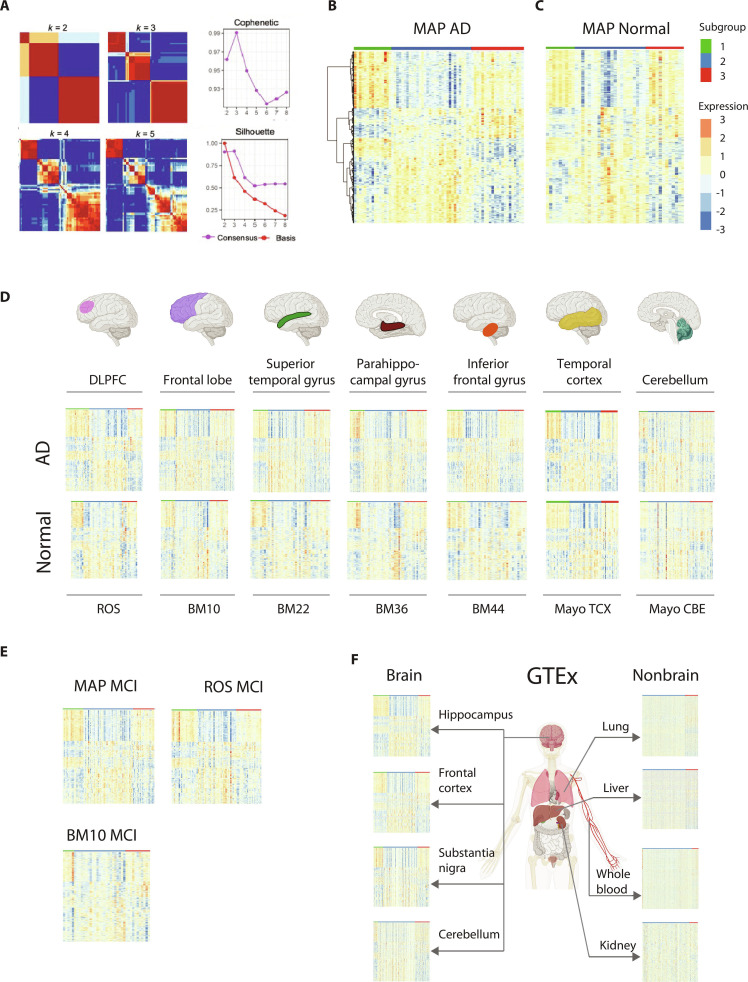
Identification and validation of a latent factor in brain RNA-seq datasets. (A) Consensus clustering matrix, cophenetic coefficient value, and silhouette value computed over 500 iterations at *k* = 2 to 8 by the Data-Adaptive Shrinkage and Clustering (DASC) algorithm. Three was chosen as the optimal *k*. (B and C) Gene expression heatmap of subgroup signatures identified through differential gene expression analysis (DEG) of the 3 subgroups in the Memory and Aging Project (MAP) RNA-seq samples of Alzheimer’s disease (AD) patients (B) and controls (C). (D) Gene expression heatmaps of the same signatures as (B) in the AD and normal samples from the Religious Orders Study (ROS) project, the Mount Sinai Brain Bank (MSBB) project, and the MayoRNAseq project. (E) Validation of the subgroup signatures in mild cognitive impairment (MCI) samples from MAP, ROS, and MSBB BM10. (F) Validation of the subgroup signatures in brain and nonbrain samples from the Genotype-Tissue Expression (GTEx) project. The sample cohorts displayed here cover various tissues and regions. MAP, ROS: dorsolateral prefrontal cortex; MB10: frontal lobe; BM22: superior temporal gyrus; BM36: parahippocampal gyrus; BM44: inferior frontal gyrus; Mayo TCX: temporal cortex; Mayo CBE: cerebellum. (Created with BioRender.com)

First, we applied DASC [12], a latent factor discovery algorithm, on an RNA sequencing (RNA-seq) dataset (*n* = 68; Table S1) derived from the dorsolateral prefrontal cortex (DLPFC) of AD patients from the Memory and Aging Project (MAP [13]) to search for latent factors that resulted in distinct subgroups of AD (Methods). We evaluated the robustness of the clusters reported by DASC (Fig. S1) and identified 3 AD subgroups (Fig. 1A). We identified cluster-specific signature genes (Table S2), defined as the significantly up-regulated genes in samples of a given cluster as compared to the rest of the samples (Methods). The subgroups displayed distinctive expression landscapes (Fig. 1B). Gene set enrichment analysis (GSEA) of the signature genes demonstrated unique enrichment terms for each subgroup: subgroup 1 was enriched in synaptic and neuronal pathways, subgroup 2 was enriched in vasculature development and oligodendrocyte differentiation, and subgroup 3 was enriched in pathways related to protein folding and antigen processing (Fig. S2). Interestingly, the latent factor did not coincide with most available clinical traits or known technical factors (Fig. S3). No significant associations were found with AD clinical or pathologic traits, such as amyloid plaques and neurofibrillary tangles, suggesting that this latent factor is unlikely to be a direct cause or consequence of the disease. Furthermore, the absence of any significant correlation between the RNA integrity number (RIN), the postmortem interval (PMI), or the age of death implies that the latent factor is unrelated to known technical confounders.

To validate this hidden factor, we tested it on over 2,000 additional brain samples across disease/control status, multiple brain regions, and multiple study cohorts. Specifically, we built a random forest classifier based on the subgroup signature genes using the MAP discovery dataset as the training data and applied it to predict subgroup labels for the validation samples (Methods). The model achieved high out-of-bag area under the receiver operating characteristic curve (AUC) for all 3 subgroups, with clear separation of predicted probabilities of MAP samples (Fig. S4).

First, we noted a remarkable similarity in the gene expression signature between MAP AD samples and MAP control samples (individuals without dementia or AD pathology; Fig. 1C). This suggests that transcriptomic differences between disease and normal states are unlikely the primary contributor to this latent factor. To determine if this latent factor reflects the inherent properties of the DLPFC or the brain more broadly, we extended our analysis to include additional RNA-seq data from various brain regions, including DLPFC from the Religious Orders Study (ROS) [14], the frontal lobe (BM10), the superior temporal gyrus (BM22), the parahippocampal gyrus (BM36), the inferior frontal gyrus (BM44) from the Mount Sinai Brain Bank (MSBB) [15], and the temporal cortex (Mayo-TCX) and the cerebellum (Mayo-CBE) from the Mayo RNA-seq Study (MayoRNAseq) [16]. The gene signature demonstrated remarkable consistency across all datasets, regardless of the disease status (Fig. 1D). Moreover, RNA-seq samples from participants with mild cognitive impairment (MCI) from MSBB, MAP, and ROS also displayed a similar expression pattern (Fig. 1E). To further investigate if this latent factor is specific to the brain, we replicated the analysis on both brain samples (hippocampus, frontal cortex, substantia nigra, and cerebellum) and nonbrain samples (lung, liver, whole blood, and kidney) from the Genotype-Tissue Expression (GTEx) project [17]. The gene signature was reproduced only in brain tissues (Fig. 1F), highlighting the brain-specific nature of the latent factor across phenotypes (AD, MCI, and normal).

### Variational spatial sampling underlies the transcriptional subgroups

**Fig. 2.**
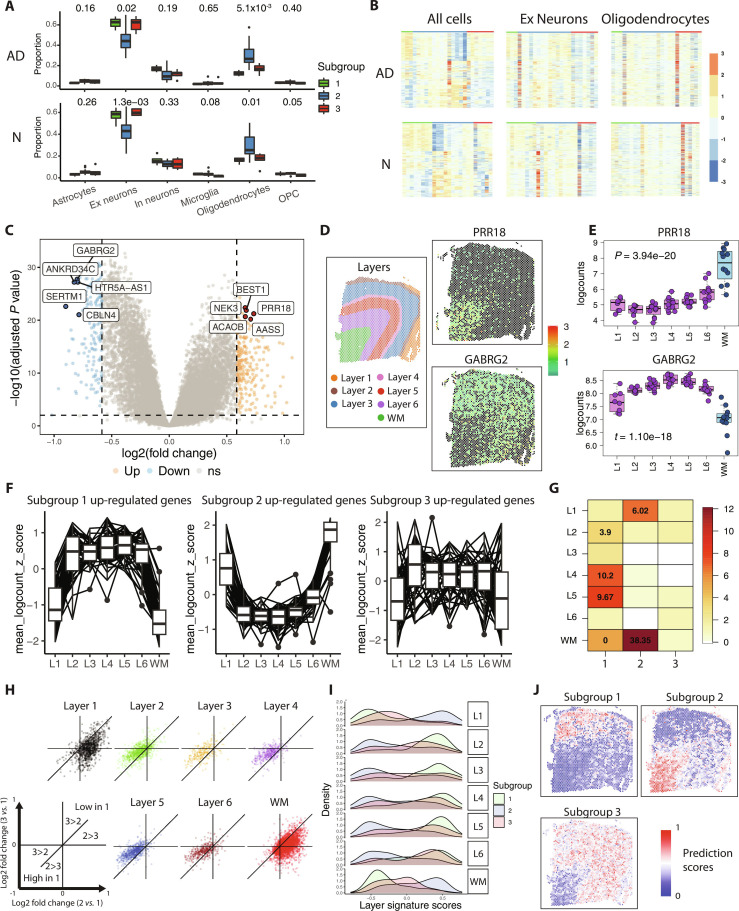
The spatial nature of the latent factor. (A) Proportions of major cell types in each subgroup in scRNA-seq samples of dorsolateral prefrontal cortex (DLPFC) of AD (*n* = 48; top panel) and normal (*n* = 24; bottom panel) participants from ROS and MAP (together referred to as ROS/MAP). The subgroup labels were obtained by applying the RF classifier on the pseudo-bulk of the scRNA-seq dataset. (B) Gene expression heatmaps of the signature genes of the pseudo-bulk data obtained by using all cells, excitatory neurons only, and oligodendrocytes only, respectively. (C) Volcano plot of differentially expressed genes in the comparison between subgroup 2 samples and the rest. The AD and normal combined samples from ROS/MAP were used. The top 5 up-regulated and down-regulated genes by log2 fold change with adjusted *P* values less than 1 × 10^−20^ were labeled. (D) Spatial distribution of *PRR18* and *GABRG2* gene expressions on a spatial transcriptomics sample of DLPFC of a normal participant available from a published study (sample ID 151673, Maynard *et al.* [19]). (E) Boxplots of *PRR18* and *GABRG2* gene expressions across cortex layers and white matter of a total of 12 spatial transcriptomics samples from the same study as (D). (F) *Z*-score normalized expressions of top 50 genes of each subgroup across cortex layers and white matter using the same data as (E). (G) Gene set enrichment of the top 50 genes of each subgroup across cortex layers and white matter using the same data as (E). (H) Pairwise comparison of log2 fold changes of layer markers between “2 *vs.* 1” and “3 *vs.* 1”. Layer markers were obtained from Maynard *et al.* [19] (I) Distribution of single sample enrichment scores of layer makers, same as (H), in each of the ROS/MAP RNA-seq samples, colored by subgroups. (J) Prediction scores of the 3 subgroups on each spatial spot of the same spatial transcriptomics data as (D). The prediction scores were *z*-score normalized.

To explore the possible origin of this latent factor, we first investigated if it could be ascribed to variations in cell-type composition. We processed single-cell RNA-seq (scRNA-seq) data of DLPFC of AD and control subjects from ROSMAP [18] and obtained an expression matrix of 17,926 genes by 75,060 nuclei (Methods). We clustered the nuclei into 6 major cell types: excitatory neurons, inhibitory neurons, astrocytes, microglia, oligodendrocytes, and oligodendrocyte progenitor cells (OPCs). We then treated the scRNA-seq data as pseudo-bulk data for each sample and applied the random forest classifier to predict the subgroup label, resulting in 11 samples assigned to subgroup 1, 25 to subgroup 2, and 12 to subgroup 3. Notably, subgroup 2 exhibited significantly lower proportions of excitatory neurons and higher proportions of oligodendrocytes (analysis of variance, *P* = 0.02 and *P* = 5.1 × 10^−3^ respectively; Fig. 2A). This cell-type proportion variation was reflected in control scRNA-seq samples (*n* = 24) from ROSMAP (Fig. 2A). We plotted the signature genes in the pseudo-bulk data (Fig. 2B) and observed a similar pattern to Fig. 1B to F. However, pseudo-bulk data based solely on a single cell type, specifically excitatory neurons or oligodendrocytes, failed to exhibit the signature pattern (Fig. 2B).

Next, we examined the most up-regulated and down-regulated genes in subgroup 2 (Fig. 2C) and found strong spatial enrichment within the gray matter (GM) and white matter (WM) regions, using an existing spatial transcriptomics dataset of the human DLPFC region [19]. For example, *PRR18*, a gene up-regulated in subgroup 2, demonstrated strong WM enrichment (WM *vs.* rest, *t* test, *P* = 3.94 × 10^−20^; Fig. 2D and E). In contrast, the gene *GABRG2*, underrepresented in subgroup 2, showed strong GM enrichment (GM *vs.* rest, *t* test, *P* = 1.10 × 10^−18^; Fig. 2D and E). We extended this analysis to include the top 50 up-regulated genes for each subgroup (Methods). Notably, subgroup 1 signature genes were predominantly abundant in layers 2 through 6, whereas subgroup 2 signature genes were more enriched in layer 1 and the WM (Fig. 2F). The distribution of subgroup 3 genes mirrored that of subgroup 1, albeit with less discernible differences between layers 2 to 6 and other layers. GSEA of the same top 50 up-regulated genes clearly showed strong enrichment of subgroup 1 genes in layers 2 to 6, subgroup 2 genes in layer 1 and WM, and moderate enrichment of subgroup 3 genes in layers 1, 2, 5, 6, and WM (Fig. 2G).

To summarize, (a) the latent factor is consistent across brain regions, (b) the latent factor is unrelated to disease, (c) subgroup 2 samples have more oligodendrocytes and fewer excitatory neurons, and (d) subgroups 1 and 3 genes are enriched in layers 2 to 6 while subgroup 2 genes are enriched in layer 1 and WM. In light of these findings, we hypothesized that the nature of this latent factor is sampling bias during tissue collection. Specifically, we hypothesized that subgroup 1 samples consisted primarily of GM, subgroup 3 samples had moderate contamination of WM, and subgroup 2 samples had substantial portions of WM. This hypothesis is supported by the observation that layers 2 to 6 markers are highest in subgroup 1 and lowest in subgroup 2, while the WM makers are highest in subgroup 2 and lowest in subgroup 1 (Fig. 2H). We then calculated single sample enrichment scores using layer-specific markers (Methods). The scores for layer 1 and WM were notably high in subgroup 2, while the scores for layers 2 to 6 were high in subgroup 1 (Fig. 2I). The distribution of those scores for subgroup 3 samples was more even (Fig. 2I). We also examined the expression of previously defined cortical layer markers [19]. Markers for layers 2 to 6 exhibited high expression in subgroup 1, low expression in subgroup 2, and moderate expression in subgroup 3 (Fig. S5). Lastly, we applied the random forest classifier on each spatial spot to predict the subgroup label. This resulted in a remarkably consistent enrichment of subgroups 1 and 3 in layers 2 to 6 and subgroup 2 in WM **(**Fig. 2J and Fig. S6).

### The latent factor transforms our understanding of AD heterogeneity

**Fig. 3.**
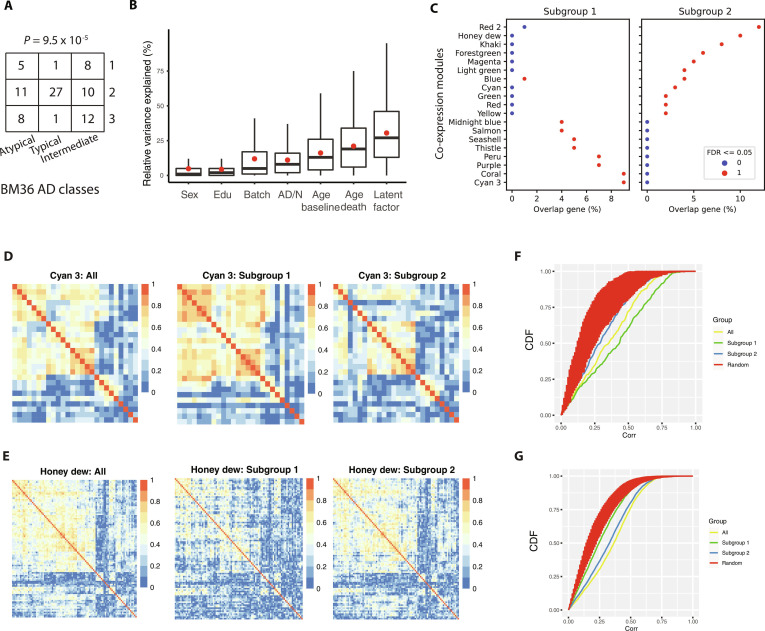
Impact of the latent factor on ROSMAP AD RNA-seq samples. (A) Confusion matrix comparing the cluster assignments using the latent factor and AD subtype clusters reported in Neff *et al.* [8] where the AD subtype clusters were divided into atypical, typical, and intermediate. Chi-square test *P* value is 9.5 × 10^−5^. (B) Variance of expression explained by clinical and technical variables and the latent factor using the ROS/MAP cohort. The difference between the latent factor and each of the other factors is significant (Wilcoxon rank-sum test, *P* < 2.0 × 10^−16^). (C) Overlap between the subgroup signature genes and the gene-regulatory modules from Zhang *et al.* [6]. Subgroup 3 was merged into subgroup 1. The *x*-axis is the percentage of module genes that overlapped with subgroup signature genes, and the *y*-axis shows the modules that are either significantly overlapped with subgroup 1 or subgroup 2 signature genes. (D and E) Heatmaps of module genes’ pairwise absolute Pearson correlation coefficients using unstratified ROSMAP samples (left), subgroup 1 samples (middle), and subgroup 2 samples (right) for the Cyan 3 (D) and Honey dew (E) modules, respectively. (F and G) Cumulative density curves of module genes’ pairwise absolute Pearson correlation coefficients using unstratified ROSMAP samples (yellow), subgroup 1 samples (green), and subgroup 2 samples (blue), and randomly sampled gene sets (red, repeat = 100) for the Cyan 3 (D) and Honey dew (E) modules, respectively. sex: gender; edu: education level; batch: RNA-seq batch; AD/N: diagnosis of AD; age baseline: baseline age; age death: age at death.

GM and WM are distinct histological regions. To assess how this latent factor would affect our understanding of AD heterogeneity, we first overlaid the clusters on the most recently identified AD subtypes using the MSBB BM36 dataset [8]. The authors identified 3 AD classes, namely, Atypical, Intermediate, and Typical. We found a significant association between the AD classes and our latent clusters (chi-square test, *P* = 9.5 × 10^−5^; Fig. 3A and Methods). Strikingly, 93% (27/29) of the Typical AD samples were cluster 2, which we identify as a WM-enriched group. The Typical subtype was enriched for myelin-related processes, vascular biology, and immune functions, consistent with our observation that cluster 2 is characterized by oligodendrocyte differentiation, blood vessel morphogenesis, and cytokine-related pathways (Fig. S2). Thus, we anticipate an improved characterization of AD subtypes by first classifying those large RNA-seq cohorts into groups enriched in either WM or GM.

We next found that the latent factor accounts for a significantly higher average explained variance of gene expression levels than age, sex, education, AD diagnosis status, and batch factors (Wilcoxon rank-sum test, *P* ≤ 2.0 × 10^−16^; Fig. 3B). Similar observations were made in the MSBB cohorts, wherein the latent factor accounts for larger variance than sex, race, and AD diagnosis, but not batch (Fig. S7). We then asked whether the latent factor would interact with AD regulatory networks, which commonly function within certain cellular communities rather than uniformly across the entire tissue [6,7,20]. We matched our signature genes with AD coexpression modules derived from MSBB bulk RNA-seq data [6] (Methods). Since subgroup 1 and subgroup 3 tend to have similar gene expression patterns (Fig. 1B) and cell-type compositions (Fig. 2A), likely reflecting GM-enriched samples, we combined them into subgroup 1. Subgroup 1 signature genes (*n* = 186) significantly overlapped with 9 modules (Fig. 3C), including synaptic transmission-related modules (Table S3). Conversely, subgroup 2 signature genes (*n* = 228) significantly overlapped with 11 modules, covering a greater diversity of categories, including GABA biosynthesis, immune functions, and nerve ensheathment (Table S3). We examined the coexpression patterns of individual modules listed above within each subtype and observed substantial subtype differences (Methods). For example, using the MSBB MB22 samples, we found that the coexpression pattern of the Cyan 3 module genes was stronger in subgroup 1, while the Honey dew module was stronger in subgroup 2 (Fig. 3D to G). We also observed significant overlaps of another set of AD modules [5], derived from ROSMAP datasets, with our subgroup signature genes (Fig. S8).

### Controlling the latent factor enhances the detection of AD-associated signature genes

**Fig. 4.**
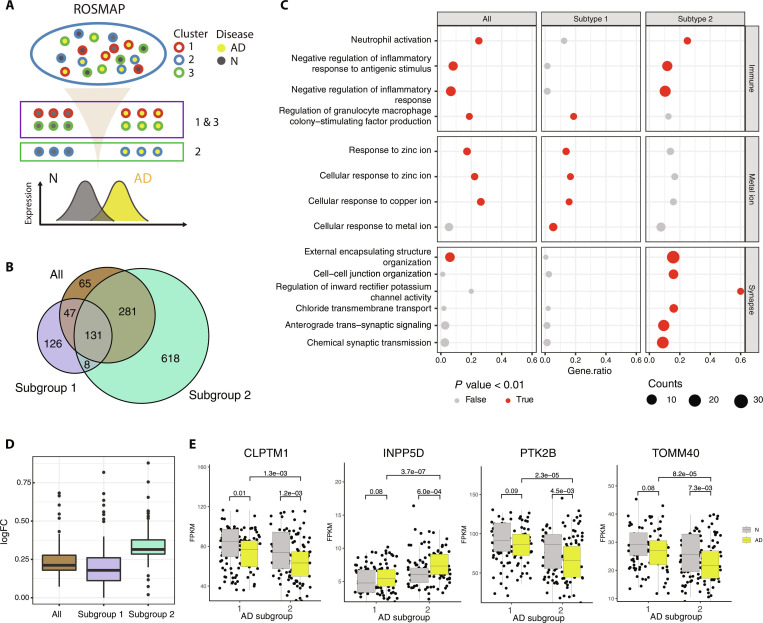
Cluster-specific differentially expressed genes (DEGs) in AD. (A) Schematic representation of the workflow used to identify DEGs between AD and normal samples of ROSMAP for each cluster. Because of the similarity of the gene expression profiles and cell-type compositions between clusters 1 and 3, samples of the 2 clusters were combined together as subgroup 1. Cluster 2 samples were termed as subgroup 2. (B) Venn diagram displaying the overlap of DEGs. We identified 312 DEGs in subgroup 1 and 1,038 DEGs in subgroup 2. The traditional way, which merged all samples of the ROSMAP cohort, identified 524 DEGs. (C) Enriched Gene Ontology (GO) terms identified using the 3 categories of DEGs in (B). Significant terms with a *P* value < 0.01 were highlighted in red. The size of the dots represents the number of DEG genes that were members of the corresponding GO terms displayed on the *y*-axis. The *x*-axis is the percentage of DEGs genes. (D) Log2 fold changes of genes in the chemical synaptic transmission term (GO: 0007268) in AD *vs.* normal. (E) Boxplot of gene expressions, in FPKM, of 4 TWAS genes (Fig. S11) that showed subgroup-specific differential patterns. *P* values calculated by the Wilcoxon signed-rank test were provided for each comparison including AD *vs.* normal, normal *vs.* normal, and AD *vs.* AD.

We performed differential gene expression analysis between normal and AD samples using the ROSMAP datasets (Methods and Fig. 4A) and found that subgroup-stratified analysis enabled recovery of nearly 3-fold more differentially expressed genes (DEGs) than a joint analysis not accounting for subgroups (Fig. 4B and Fig. S10). This indicates that accounting for unappreciated spatial heterogeneity provided a higher resolution in characterizing AD transcriptional abnormalities. We also tested whether including the WM enrichment score as a covariate in the limma model could achieve comparable DEG detection. The RF-derived WM score, though not an absolute WM fraction, is significantly correlated with ssGSEA scores computed from WM-marker genes (Spearman *ρ* = 0.563, *P* < 2.2 × 10^−16^; Fig. S9 and Methods), validating it as a continuous proxy for WM content. This model generated slightly higher DEG yield than the default model (Table S4). Gene Ontology (GO) enrichment analysis showed that the top enriched GO terms in the DEGs from the joint analysis were also significantly enriched in subgroup-specific DEGs, with subgroups 1 and 2 behaving differently (Fig. 4C). For example, subgroup 1 DEGs are enriched in pathways of cellular responses to metal ions, while subgroup 2 DEGs were enriched in immune function pathways and synaptic transmission. In addition to the differential biological pathways, the effect sizes of the DEGs were also substantially different among subgroups. Using the genes from the pathways significant only in subgroup 2 (Fig. 4C, bottom panel), we demonstrated higher log2 fold changes of the DEGs in subgroup 2 than in subgroup 1 (Fig. 4D). We searched for potential AD driver genes using transcriptome-wide association study (TWAS) with AD as the phenotype of interest [21] (Fig. S11 and Methods) and identified a few, including *CLPTM1*, *INPP5D*, *PTK2B*, and *TOMM40*, that displayed subgroup-specific differential expression between AD and normal samples (Fig. 4E). There results strongly suggested that controlling for the latent factor can enhance the identification of transcriptional abnormalities and direct focused research to specific brain regions.

### Identification of the same latent factor in the metabolome and proteome of the human brain

**Fig. 5.**
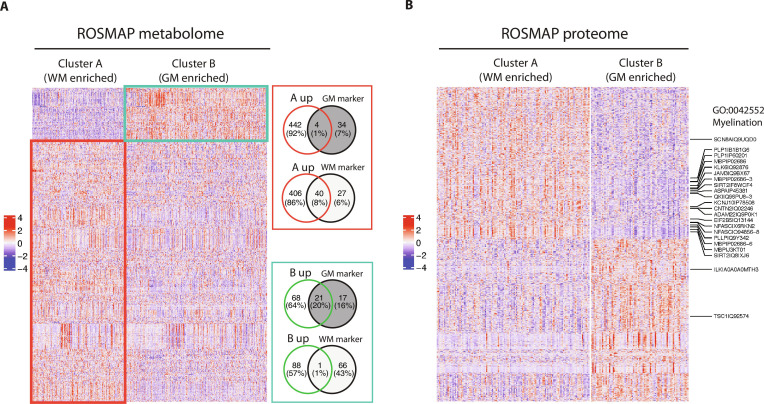
Detection of GM- and WM-enriched clusters in ROS/MAP metabolome and proteome. (A) Heatmap of metabolites for 2 metabolome clusters identified with DASC in 514 ROS/MAP samples, and Venn plots displaying the overlap of cluster signature metabolites with GM and WM markers derived from a published dataset. (B) Heatmap of protein expressions for 2 clusters identified with DASC in 208 ROS/MAP samples. Significant differential proteins in the myelination pathways were labeled.

We anticipated the presence of similar sampling bias in human brain samples profiled for other molecular types. We tested this idea on the metabolome (sample size *n* = 514) and proteome (*n* = 208) data of ROSMAP DLPFC cohorts for which we obtained 2 subgroups using DASC (Methods and Fig. 5A and B). We derived metabolite markers for GM and WM from a published study (Methods) where 540 metabolites were quantified in 158 participants [22]. Metabolites enriched in subgroup A (Table S5) strongly overlapped with WM markers (Table S6), while those enriched in subgroup B overlapped with GM markers (Fig. 5A). For the proteome data, the most significantly up-regulated pathway in cluster A based on the differential proteins (Table S7) is Ensheathment of Neurons (GO:0007272; Fig. S12), a process in which glial cells envelop neuronal cell bodies and axons. This process can take the form of myelinating or nonmyelinating ensheathment (myelin gives the WM its color and is mainly formed by oligodendrocytes, a type of glial cell). Myelination-related proteins are almost exclusively up-regulated in cluster A of the proteome data (Fig. 5B). Both the metabolome and proteome clustering patterns clearly suggest the existence of sampling bias that leads to both WM-enriched and GM-enriched samples in the cohorts.

## Discussion

This work highlighted a latent factor in brain transcriptome, metabolome, and proteome datasets and presumably in other data types. We revealed that this latent factor was attributable to sampling bias that led to the over- or underrepresentation of the GM and WM in the samples. Accounting for this sampling bias holds a strong potential to improve our understanding of the brain and neurological disorders, considering the intrinsic molecular, cellular, and functional differences between WM and GM. Traditional analyses of neurological disorders, including AD, have been hindered by diminished signals due to such sampling bias. We demonstrated that, by accounting for this latent factor, we identified 2.6 times more DEGs in AD and revealed the region specificity of AD genes. We also showed a strong confounding effect of this latent factor in AD subtyping. Consideration of the latent factor will enhance molecular subtyping of AD in the future, advancing efforts to develop more targeted diagnostic and therapeutic strategies.

Our findings also have practical implications for study design and data analysis. Because this latent factor most likely reflects variation in the relative contribution of GM and WM, greater attention to tissue sampling procedures, anatomical matching, and collection of detailed sampling metadata may help reduce this source of bias in future studies. For existing datasets, the latent factor or a derived proxy score can be used either to stratify samples into more comparable groups or to adjust for tissue composition effects in downstream analyses.

A limitation of the current study is that direct quantitative estimates of WM and GM content were not available for bulk samples. Although multiple lines of evidence support our interpretation that this latent factor reflects variation in relative WM and GM material, this conclusion remains inferential rather than directly measured. Future studies with direct measurements of GM and WM composition in the same samples will be important to further validate this interpretation.

## Methods

### Bulk RNA-seq data

We used brain RNA-seq data from 4 studies for discovery and validation, namely, ROS, MAP, MSBB, and MayoRNAseq, all coming from the AMP-AD project. The AD, normal, and MCI samples were identified based on specific selection criteria. For ROS and MAP, AD: clinical consensus diagnosis (cogdx) = 4, Braak stage ≥4, the Consortium to Establish a Registry for AD (CERAD) score ≤ 2; normal: cogdx = 1, Braak stage ≤ 3, and CERAD ≥ 3; MCI: cogdx = 2, 3. For MSBB, AD: clinical dementia rating (CDR) ≥ 1, neuropathology category as measured by CERAD (NP.1) ≥ 2, and Braak score ≥ 4; normal: CDR ≤ 0.5, NP.1 ≤ 1, and Braak score ≤ 3; MCI: CDR = 0.5. For MayoRNAseq, we used the diagnosis labels provided by the original study [16].

ROS and MAP (collectively known as ROSMAP [23]) samples were extracted from the DLPFC of the brain. From ROS, we obtained 75 AD, 43 normal, and 75 MCI samples. Similarly, from MAP, we obtained 68 AD, 43 normal, and 94 MCI samples. The MSBB dataset comprises 4 cohorts: BM10 of the frontal lobe, BM22 of the superior temporal gyrus, BM36 of the parahippocampal gyrus, and BM44 of the inferior frontal cortex. We obtained 191 AD, 35 normal, and 39 MCI samples from BM10; 200 AD and 32 normal samples from BM22; 194 AD and 36 normal samples from BM36; and 166 AD and 27 normal samples from BM44. The MayoRNAseq cohort consists of 82 AD and 79 normal samples from the cerebellum and 82 AD and 80 normal samples from the temporal cortex. Additionally, we obtained RNA-seq data from the GTEx project for the following tissues: brain frontal cortex BA9 (*n* = 209), brain substantia nigra (*n* = 139), brain hippocampus (*n* = 197), brain cerebellum (*n* = 241), kidney (*n* = 85), lung (*n* = 578), liver (*n* = 226), and whole blood (*n* = 755).

### Discovery of the latent factor

First, we filtered the discovery dataset (MAP) by removing the Y chromosome genes to reduce the sex effect. We removed transcripts with low counts, resulting in a total of 17,538 genes. We normalized the gene counts using DESeq2 [24] and applied log2 transformation to the counts. We corrected for batches, RIN, and PMI, using the limma package [25] function “removeBatchEffect”. The same workflow was applied to other cohorts. We applied the DASC [12] algorithm on the cleaned expression data with the number of clusters ranging from *k* = 2 to *k* = 8, with 500 iterations along with default settings. DASC starts by obtaining the batch-free matrix U (biological signal) using a nonparametric data-adaptive shrinkage method. The batch-free matrix is then used to obtain the batch-matrix B. Semi-NMF is then applied to the batch matrix B to find hidden batch factors.

To assess the robustness of the DASC subgroup assignments, we performed a sensitivity analysis over a grid of algorithm parameters. DASC was applied to MAP AD samples (*n* = 68) across 3 feature-selection thresholds (top 2,000, 3,000, or 4,000 genes by median absolute deviation), 3 regularization parameters (*λ* = 0.001, 0.01, and 0.1), and 3 NMF consensus iteration counts (10, 100, and 500), yielding 9 parameter combinations per iteration count. For each combination, cluster assignments were obtained at ranks *k* = 2 through 8. Pairwise adjusted Rand index scores were then computed between all 9 parameter settings at each rank and iteration count to quantify the concordance of cluster assignments across parameter choices.

### Signature genes

We identified DEGs for each subgroup by comparing the samples of a given subgroup against the remaining samples using DESeq2 [24] with default settings. We then selected the top DEGs that passed a false discovery rate (FDR) of 1% and had an absolute fold change ≥1.5. This method yielded a gene signature of 2,252 genes.

### Gene set enrichment analysis

GSEA was done using fgsea [26] v1.12.0 in R. We selected the C2 Kyoto Encyclopedia of Genes and Genomes (KEGG) subset (186 gene sets) and C5 GO subset (15,937 gene sets) obtained from MSigDB (https://www.gsea-msigdb.org/gsea/msigdb/index.jsp). Input genes were ranked based on log fold change in each subgroup. Parameters used in GSEA are as follows: max size = 500; min size = 10; number of permutations = 10,000. We selected significantly enriched gene sets with adjusted *P* value less than 0.01 and effect size larger than 0. Top gene sets from different sources (KEGG and GO) were merged and plotted.

### Random forest classifier

We built a random forest classifier with 200 trees using the 2,252 genes using the MAP discovery dataset. The random forest classifier was then used to predict a subgroup label for each sample in the validation studies. When plotting the heatmap for the validation studies, we used the same gene order observed from the MAP AD study. Model performance was evaluated using out-of-bag (OOB) estimation from the random forest model. OOB accuracy served as an internal measure of classification performance, and per-class one-*vs.*-rest AUC was computed to assess class-specific discrimination.

### Single-sample GSEA

We obtained WM signature genes from the spatial transcriptomics study [19] by selecting genes with *t* statistics > 10 in WM as compared to other layers, yielding 302 genes (299 present in the expression matrix). Single-sample GSEA (ssGSEA) was performed on the ROSMAP normalized expression matrix using the gene set variation analysis (GSVA) R package [27] to compute a per-sample WM enrichment score. The correlation between the ssGSEA-derived WM score and the RF-predicted subgroup 2 (WM) probability was assessed using both Spearman and Pearson correlation tests, computed for the pooled cohort.

### scRNA-seq analysis

We obtained scRNA-seq data of 72 samples, consisting of 48 AD and 24 control samples, from the ROSMAP cohort [28] and followed methods of the original study to obtain the gene expression matrix of 17,926 genes in 75,060 nuclei. Normalization and clustering were performed with Seurat v3 [29]. Counts for all nuclei were scaled by the total library size multiplied by 10,000 and transformed to log space. A total of 3,000 top highly variable genes were identified using Seurat to perform principal component analysis (PCA). The top 10 PCs were used to build the k-nearest neighbors (KNN) cell–cell graph with *k* = 20. Then, the shared nearest neighbor was built based on the KNN graph, in which clusters of the cells are identified. We inferred the cell types for each cluster using cell-type markers reported in Lake *et al.* [30]. We ended up with 6 main cell types, including excitatory neurons (49.9%), inhibitory neurons (12.4%), astrocytes (4.8%), microglia (2.7%), oligodendrocytes (25.8%), and OPCs (3.6%).

We transformed the scRNA-seq expression data to a pseudo-bulk for each sample. We then applied the random forest classifier to predict the subgroup labels as we did for the bulk RNA-seq data. The expressions of 1,732 genes that were captured in the scRNA-seq data were used.

### Spatial transcriptomics data analysis

We downloaded 12 human DLPFC samples [19] using the R package *SpatialLIBD* [19,31]*.* In total, we obtained 47,681 spatial domains with transcriptome sequenced across the 12 DLPFC tissues. We used the functions provided in the SpatialLIBD package to visualize the spot-level and layer-level gene expressions. The top 50 significantly up-regulated genes with an average expression above 8, ranked by log2 fold changes, of each subgroup were used to calculate layer enrichment scores. Median log-counts of each gene were calculated and *z*-score normalized across layers to examine the distribution patterns.

Layer-specific up-regulated genes were obtained from the same study. Genes with a *t* statistic greater than 5 and a *P* value smaller than 0.01 were extracted for each layer against the rest. We used the GSVA package [27] to calculate a single sample enrichment score using the layer-specific up-regulated genes for AD and normal samples of the ROSMAP dataset.

We applied the pretrained random forest model to predict the probability scores of the subgroups for the spatial spots of the 12 samples. Gene expression values were count-per-million normalized and log-scaled to account for different sequencing depths and reduce the impact of outliers. The classifier was then applied to calculate the subgroup probability scores.

### Overlap between previously identified AD subtypes and the latent clusters

We obtained the AD subtype labels for MSBB BM36 samples from Neff *et al.* [8]. The number of samples common with the BM36 samples we analyzed was 83. We applied the random forest classifier to predict the cluster labels and applied chi-square test to test the association between the AD subtypes and the latent clusters.

### The proportion of variance explained by clinical variables and the latent factor

We used ROSMAP and MSBB RNA-seq data to estimate the proportion of variance explained by the latent factor. Raw read counts were library normalized and log-scaled to adjust for different sequencing depths and reduce the impact of outliers. Then, the expression vector for each gene is further normalized to have a mean of zero and a standard deviation of one. Clinical and technical factors were not adjusted in the expression matrix for this analysis. For each gene, the variance explained by the latent factor label and each of the 6 variables, including sex, education, age of initial visit, age of death, batch, and Alzheimer diagnosis, are quantified by fitting a linear mixed model using the R package variancePartition [32] for the ROSMAP dataset. For MSBB, sex, race, batch, and diagnosis were included. Proportions of variances explained by each factor were calculated after removing the residues for each gene. The top 9,000 highly variable genes were used for visualization and comparison.

### Overlap between subgroup DEGs and known AD module genes

Samples predicted as subgroups 1 and 3 are grouped as subgroup 1. Then, DEGs with fold changes ≥ 50% and adjusted *P* < 0.05 for each subgroup are extracted. As a result, we obtained 186 DEGs for subgroup 1 and 228 DEGs for subgroup 2. Then, we tested the significance of overlap between subgroup-specific DEGs and 2 sets of published AD modules, one derived from MSBB [6] and the other from ROSMAP [5], using hypergeometric test by calling the function scipy.stats.hypergeom().

### Intra-module gene coexpression analysis stratified by subgroups

First, our pretrained random forest model stratified the samples by assigning them to either subgroup 1 or subgroup 2. For genes belonging to each module, we calculated the pairwise gene–gene expression values’ Pearson correlation coefficients using unstratified MSBB BM22 samples and stratified subgroup 1 and subgroup 2 samples. Additionally, we randomly sampled a gene set the same size as the module and calculated the random gene set’s correlations as a control. The random sampling was repeated 100 times to avoid bias in a single random experiment. In the end, the correlation matrices for each subgroup, unstratified samples (all samples), and 100 random gene sets were generated for each module. Cumulative density function estimations of module genes’ absolute correlations for unstratified samples (all samples), subgroup 1, subgroup 2 and the 100 random repeats were generated to investigate the distributional differences of module genes’ coexpression patterns in the 2 subgroups and compared with random gene sets.

### DEGs between AD and normal in a subgroup-specific manner

We used limma for DEG analysis. With an adjusted *P* value of 0.05 and a fold change of 1.2 as the cutoff, we obtained 312 DEGs for subtype 1, 1,038 for subtype 2, and only 524 if combining all samples. The DEG ENSEMBL ID was converted to gene symbol by the BioTools.fr webpage tool. Next, gene symbol lists were provided to Enrichr [33] for GO GSEA. The top enriched terms of Biological Process were grouped by functions and plotted.

### DEGs between AD and normal by adjusting for the subgroup scores

To account for variability in WM tissue composition across bulk RNA-seq samples, we added the subgroup 2 prediction score from the random forest classifier as a covariate in the limma model (~ diagnosis + subgroup_2_score), so that the diagnosis coefficient estimates the AD-*vs.*-control effect at the average WM composition.

### TWAS analysis

We downloaded the summary statistics of 6 GWAS studies that searched for variants associated with AD from NHGRI-EBI GWAS Catalog (https://www.ebi.ac.uk/gwas/home) [21]. The study IDs are as follows: GCST002245, GCST005922, GCST007320, GCST007511, GCST009019, and GCST009020. These studies recruited European participants. We downloaded eQTL models from FUSION [34] at http://gusevlab.org/projects/fusion/. The models we used were built on Brain Cortex samples from GTEx v8 expression data. The linkage-disequilibrium panel inferred on European populations from the 1000G project was also obtained from FUSION. Then, the FUSION algorithm was run with the default settings to estimate the significance of genes in association with AD. TWAS *z*-score ≥ 5 and FDR ≤ 0.05 were used to select significant genes.

### Metabolome analysis

ROSMAP metabolomic data were downloaded from the AD Knowledge portal (Synapse ID: syn25878459). Brain levels of metabolites were quantified using an untargeted mass spectrometry-based platform (Metabolon HD4) followed by block correction and normalization as described on the Synapse page. Metabolites with missing values in more than 70% of samples were discarded. Missing values were imputed using random forest imputation [35]. We used 514 ROSMAP samples to identify 2 metabolome subgroups using DASC with 100 iterations. *Z*-score normalization followed by quantile normalization was performed to visualize the metabolome data.

### Metabolite markers

We downloaded metabolome data from a published study, where 540 metabolites were quantified in the GM and WM of Broadmann Area 9 of 158 individuals [22]. Fold changes of the 540 metabolites were calculated between WM and GM in 4 groups defined based on Braak stage and APOE genotypes. Metabolites with a fold change equal to or greater than 2 in at least 3 groups were considered WM or GM markers separately.

### Proteome analysis

We obtained postprocessed protein expression data from the AD Knowledge portal (Synapse ID: syn25006864). Proteins with missing values in more than 70% of samples were discarded. Missing values were imputed using random forest imputation [35]. We ran DASC with 100 iterations to classify 208 samples into 2 clusters. GSEA was performed to identify the most significantly enriched GO pathways for each cluster. *Z*-score normalization followed by quantile normalization was performed to visualize the proteome data.

## Data Availability

Codes used in this study were available on github: https://github.com/LiuzLab/AD_LatentFactor.
