# Supplementary material for: Latent Factor Modeling Reveals Unexpected Spatial Heterogeneity in Human Alzheimer’s Disease Brain Transcriptomes
Source: Comput Struct Biotechnol J. 2026 May 14;35(1):0108. doi: 10.34133/csbj.0108 (PMC13172580; doi:10.34133/csbj.0108)
Supplement: Supplementary 1 — Figs. S1 to S12 Tables S1 to S7 [file csbj.0108.f1.zip › Supplementary figures.pdf]

Figure S1.

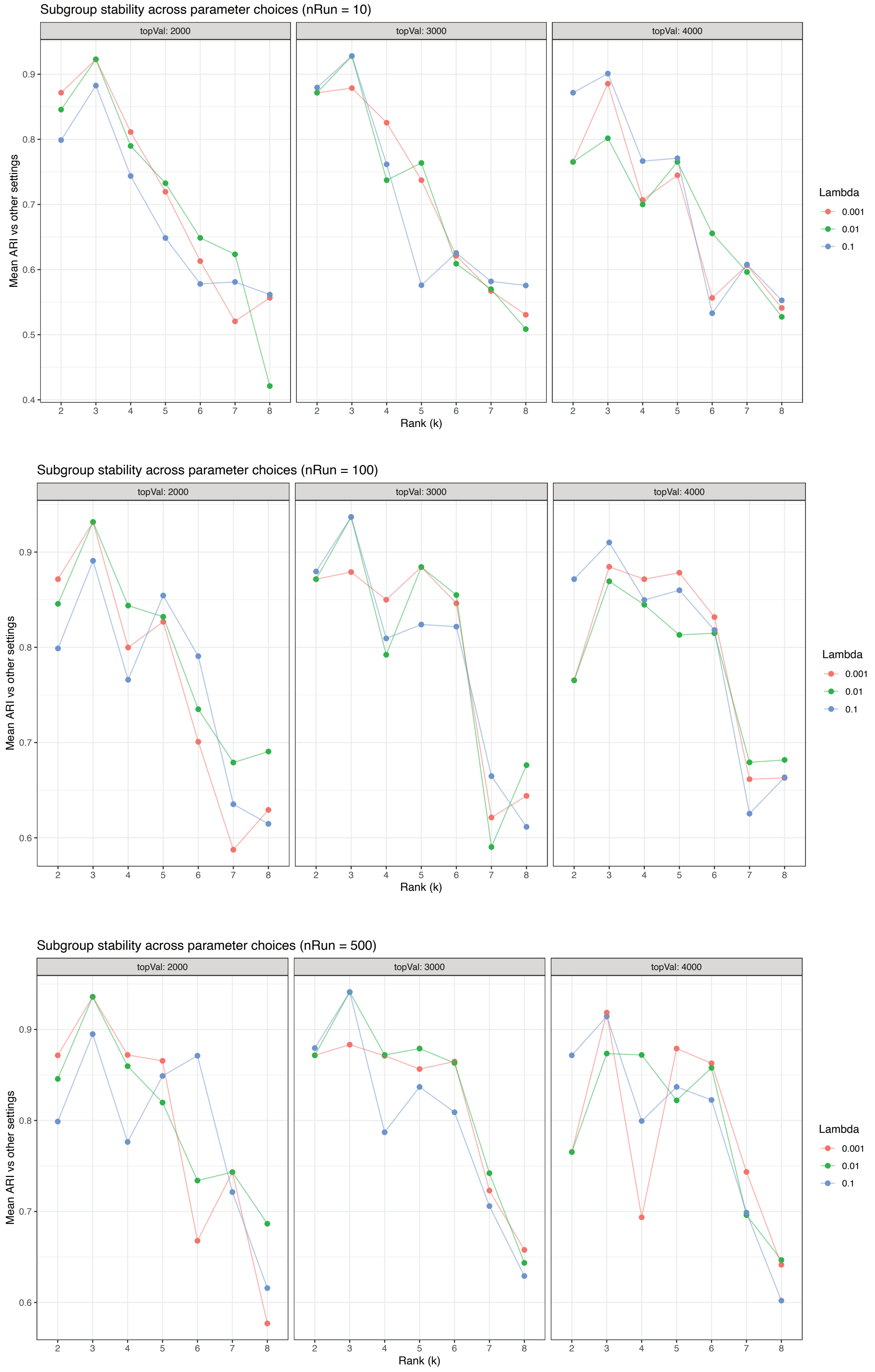
Subgroup stability across parameter choices (nRun = 10)
topVal: 2000
topVal: 3000
topVal: 4000
Mean ARI vs other settings
Rank (k)
Lambda
0.001
0.01
0.1
Subgroup stability across parameter choices (nRun = 100)
topVal: 2000
topVal: 3000
topVal: 4000
Mean ARI vs other settings
Rank (k)
Lambda
0.001
0.01
0.1
Subgroup stability across parameter choices (nRun = 500)
topVal: 2000
topVal: 3000
topVal: 4000
Mean ARI vs other settings
Rank (k)
Lambda
0.001
0.01
0.1

Figure S2.

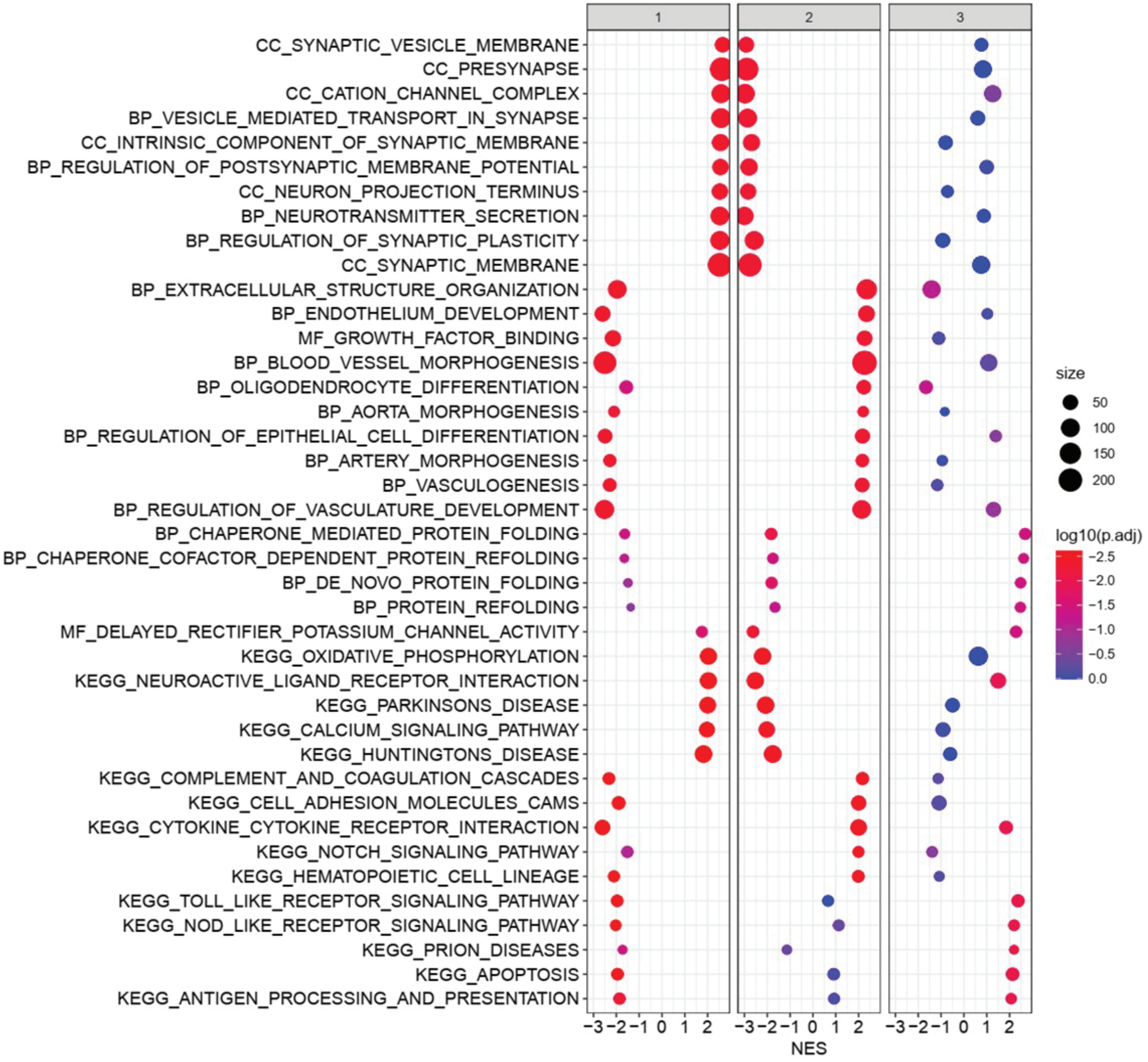

1
2
3
CC_SYNAPTIC_VESICLE_MEMBRANE
CC_PRESYNAPSE
CC_CATION_CHANNEL_COMPLEX
BP_VESICLE_MEDIATED_TRANSPORT_IN_SYNAPSE
CC_INTRINSIC_COMPONENT_OF_SYNAPTIC_MEMBRANE
BP_REGULATION_OF_POSTSYNAPTIC_MEMBRANE_POTENTIAL
CC_NEURON_PROJECTION_TERMINUS
BP_NEUROTRANSMITTER_SECRETION
BP_REGULATION_OF_SYNAPTIC_PLASTICITY
CC_SYNAPTIC_MEMBRANE
BP_EXTRACELLULAR_STRUCTURE_ORGANIZATION
BP_ENDOTHELIUM_DEVELOPMENT
MF_GROWTH_FACTOR_BINDING
BP_BLOOD_VESSEL_MORPHOGENESIS
BP_OLIGODENDROCYTE_DIFFERENTIATION
BP_AORTA_MORPHOGENESIS
BP_REGULATION_OF_EPITHELIAL_CELL_DIFFERENTIATION
BP_ARTERY_MORPHOGENESIS
BP_VASCULOGENESIS
BP_REGULATION_OF_VASCULATURE_DEVELOPMENT
BP_CHAPERONE_MEDIATED_PROTEIN_FOLDING
BP_CHAPERONE_COFACTOR_DEPENDENT_PROTEIN_REFOLDING
BP_DE_NOVO_PROTEIN_FOLDING
BP_PROTEIN_REFOLDING
MF_DELAYED_RECTIFIER_POTASSIUM_CHANNEL_ACTIVITY
KEGG_OXIDATIVE_PHOSPHORYLATION
KEGG_NEUROACTIVE_LIGAND_RECEPTOR_INTERACTION
KEGG_PARKINSONS_DISEASE
KEGG_CALCIUM_SIGNALING_PATHWAY
KEGG_HUNTINGTONS_DISEASE
KEGG_COMPLEMENT_AND_COAGULATION_CASCADES
KEGG_CELL_ADHESION_MOLECULES_CAMS
KEGG_CYTOKINE_CYTOKINE_RECEPTOR_INTERACTION
KEGG_NOTCH_SIGNALING_PATHWAY
KEGG_HEMATOPOIETIC_CELL_LINEAGE
KEGG_TOLL_LIKE_RECEPTOR_SIGNALING_PATHWAY
KEGG_NOD_LIKE_RECEPTOR_SIGNALING_PATHWAY
KEGG_PRION_DISEASES
KEGG_APOPTOSIS
KEGG_ANTIGEN_PROCESSING_AND_PRESENTATION
−3 −2 −1 0 1 2
NES
size
50
100
150
200
log10(p.adj)
−2.5
−2.0
−1.5
−1.0
−0.5
0.0

Figure S3

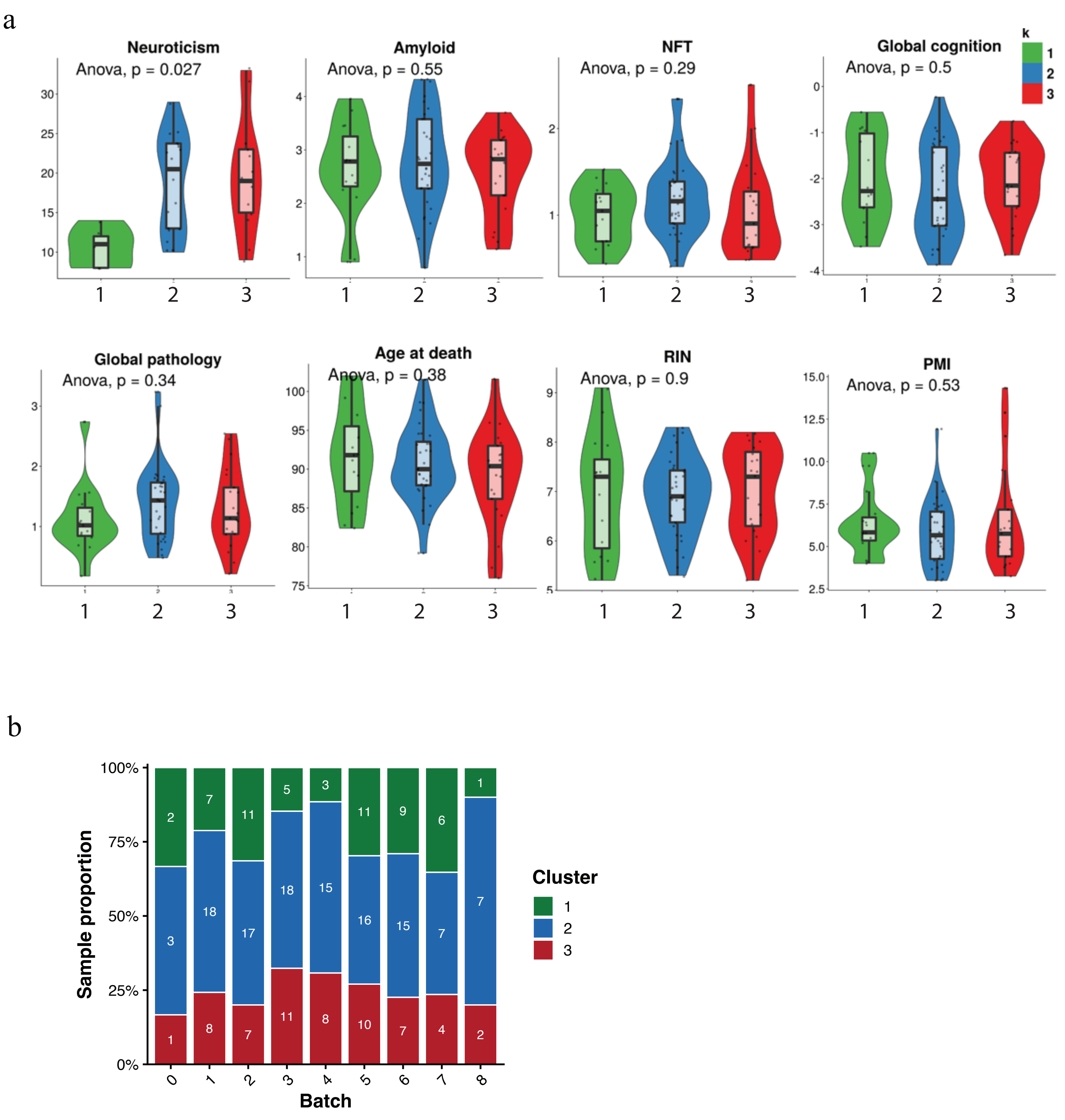

a
Neuroticism
Anova, p = 0.027
Amyloid
Anova, p = 0.55
NFT
Anova, p = 0.29
Global cognition
Anova, p = 0.5
k
1
2
3
Global pathology
Anova, p = 0.34
Age at death
Anova, p = 0.38
RIN
Anova, p = 0.9
PMI
Anova, p = 0.53
b
Sample proportion
100%
75%
50%
25%
0%
Batch
0 1 2 3 4 5 6 7 8
2 7 11 5 3 11 9 6 1
3 18 17 18 15 16 15 7 7
1 8 7 11 8 10 7 4 2
Cluster
1
2
3

Figure S4.

a

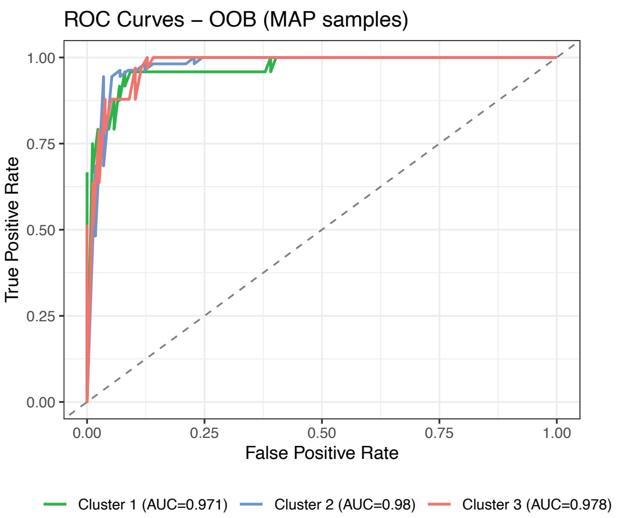

ROC Curves – OOB (MAP samples)
True Positive Rate
False Positive Rate
0.00
0.25
0.50
0.75
1.00
Cluster 1 (AUC=0.971)
Cluster 2 (AUC=0.98)
Cluster 3 (AUC=0.978)

b

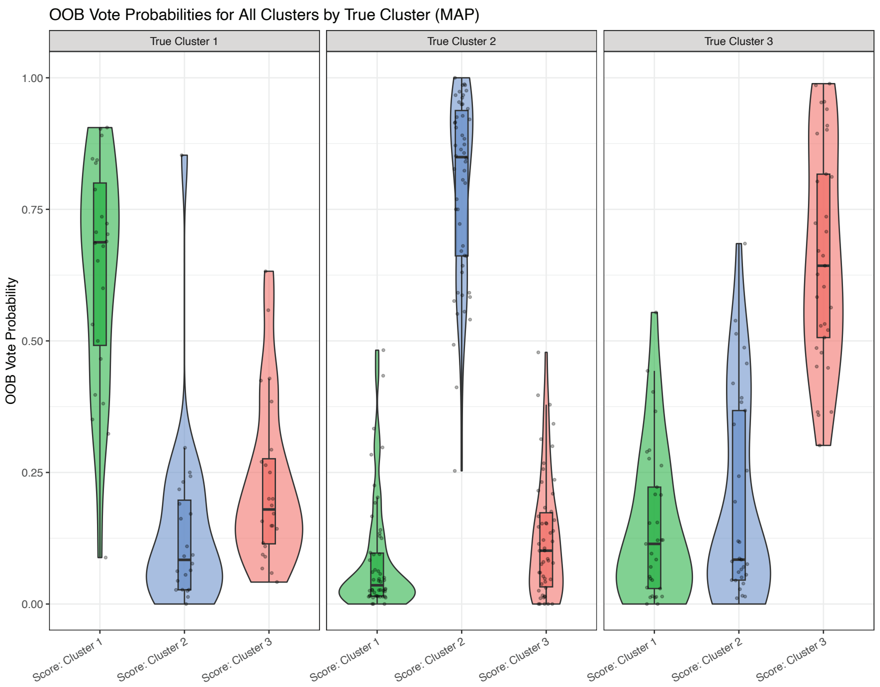

OOB Vote Probabilities for All Clusters by True Cluster (MAP)
True Cluster 1
True Cluster 2
True Cluster 3
OOB Vote Probability
0.00
0.25
0.50
0.75
1.00
Score: Cluster 1
Score: Cluster 2
Score: Cluster 3

Figure S5.

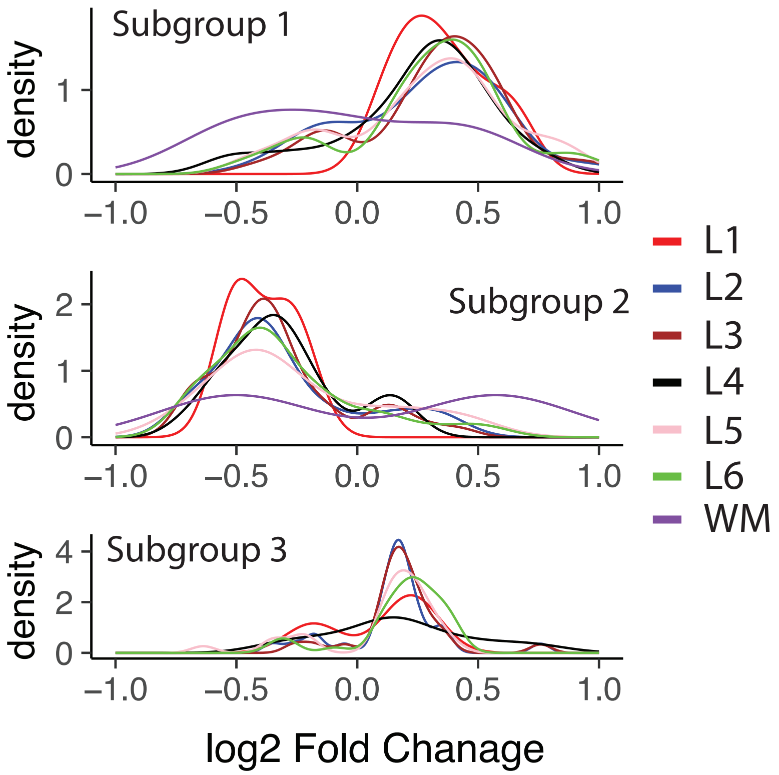

Subgroup 1
density
1
0
−1.0
−0.5
0.0
0.5
1.0
Subgroup 2
density
2
1
0
−1.0
−0.5
0.0
0.5
1.0
Subgroup 3
density
4
2
0
−1.0
−0.5
0.0
0.5
1.0
log2 Fold Chanage
L1
L2
L3
L4
L5
L6
WM

Figure S6.

Participant 1:

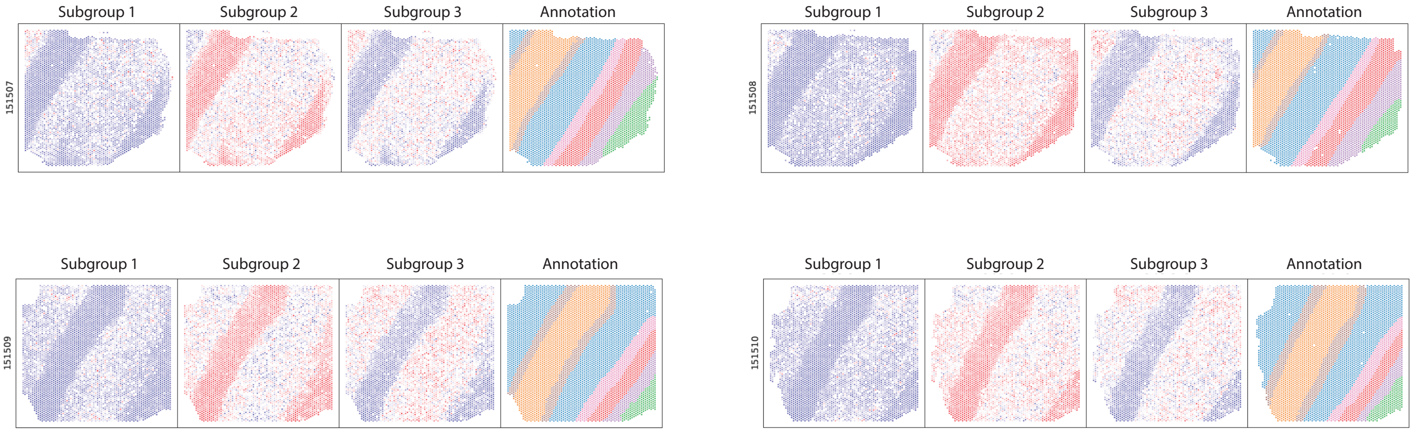

Participant 2:

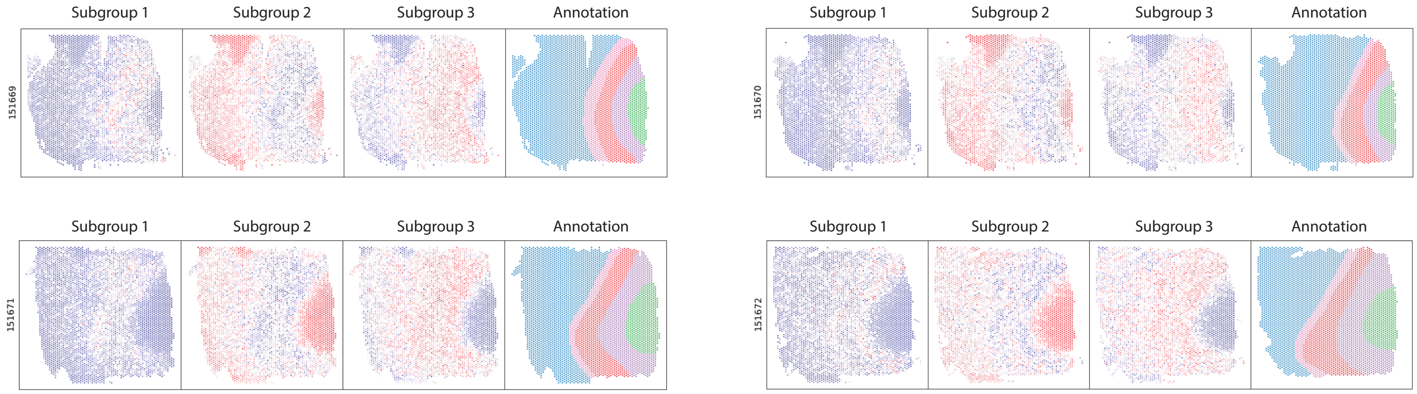

Participant 3:

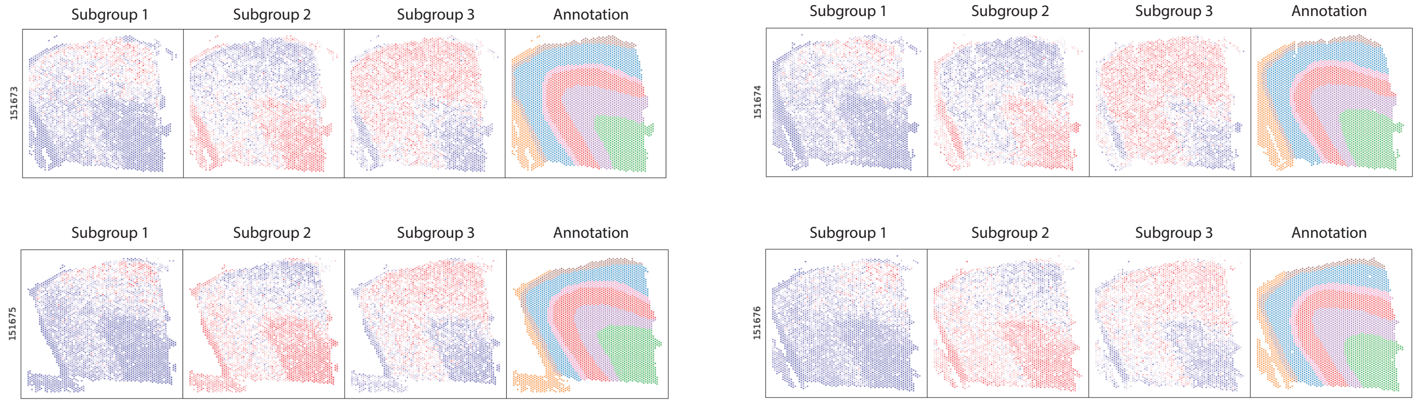

Figure S7.

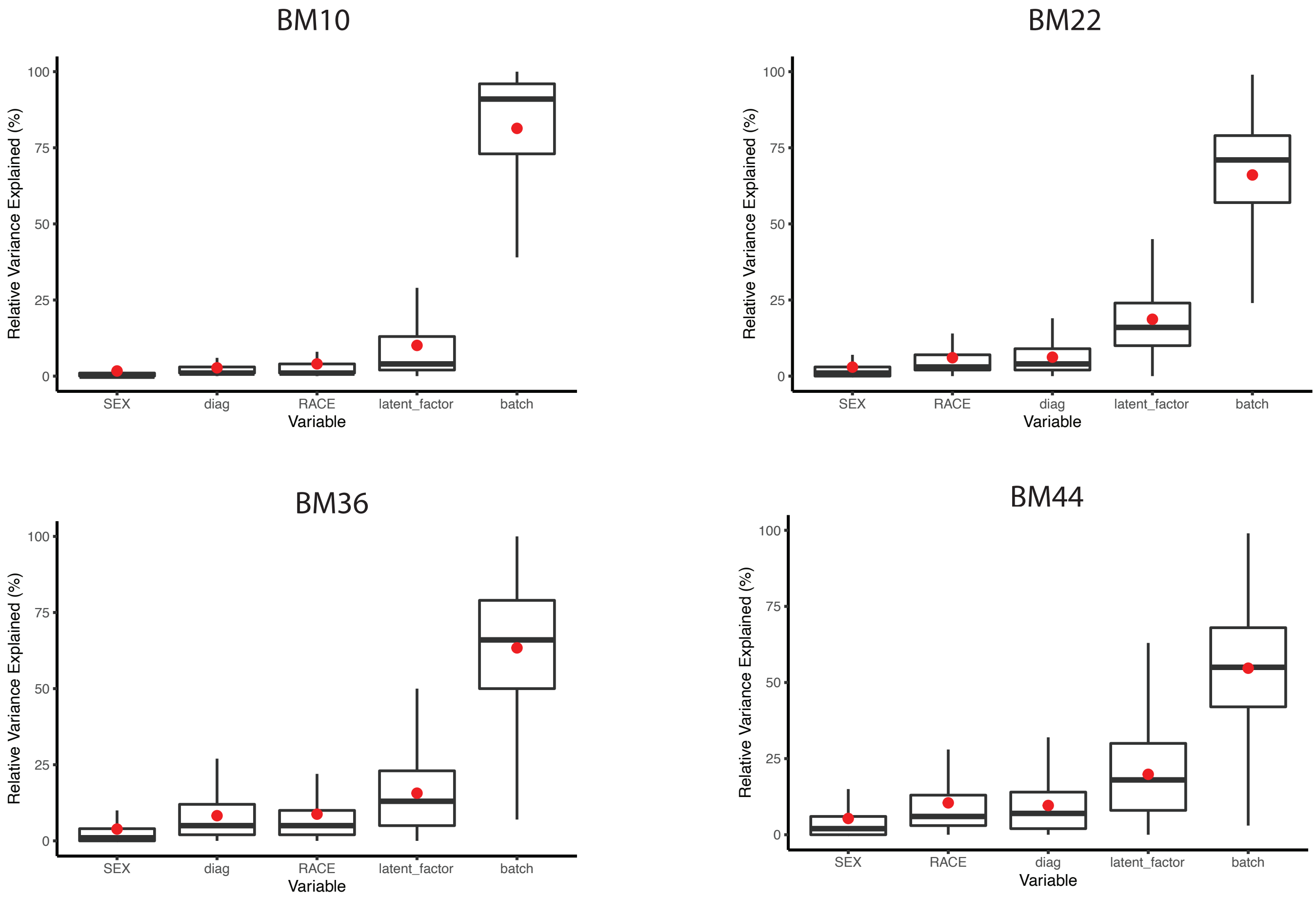
BM10
BM22
BM36
BM44
Relative Variance Explained (%)
100
75
50
25
0
SEX
diag
RACE
latent_factor
batch
Variable

Figure S8

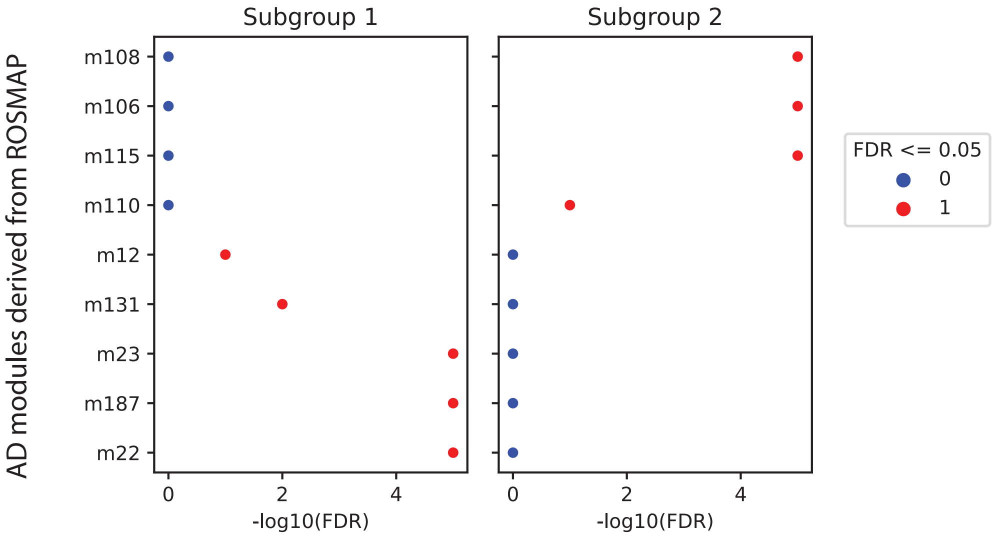
Subgroup 1
Subgroup 2
AD modules derived from ROSMAP
m108
m106
m115
m110
m12
m131
m23
m187
m22
0
2
4
-log10(FDR)
FDR <= 0.05
0
1

Figure S9.

a

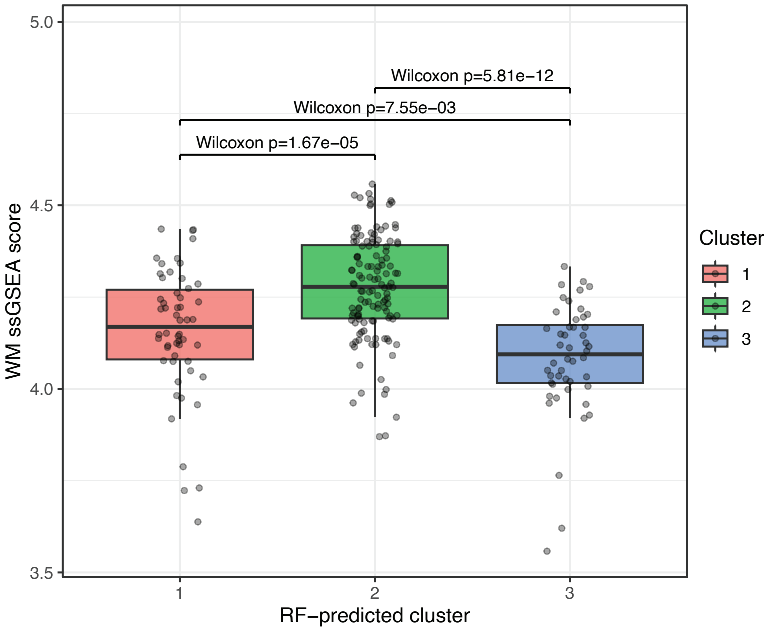

Wilcoxon p=5.81e−12
Wilcoxon p=7.55e−03
Wilcoxon p=1.67e−05
WM ssGSEA score
5.0
4.5
4.0
3.5
1
2
3
RF−predicted cluster
Cluster
1
2
3

b

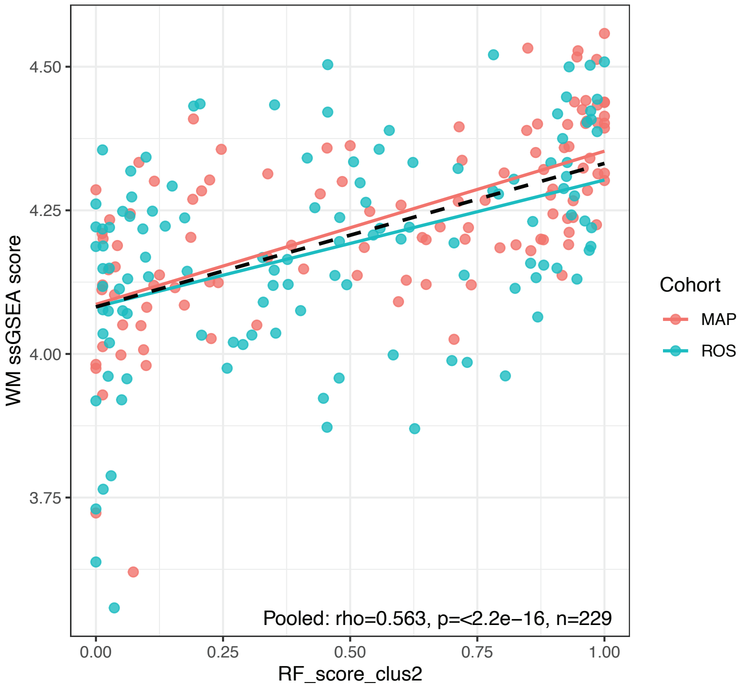

WM ssGSEA score
4.50
4.25
4.00
3.75
0.00
0.25
0.50
0.75
1.00
RF_score_clus2
Pooled: rho=0.563, p=<2.2e−16, n=229
Cohort
MAP
ROS

Figure S10.

a

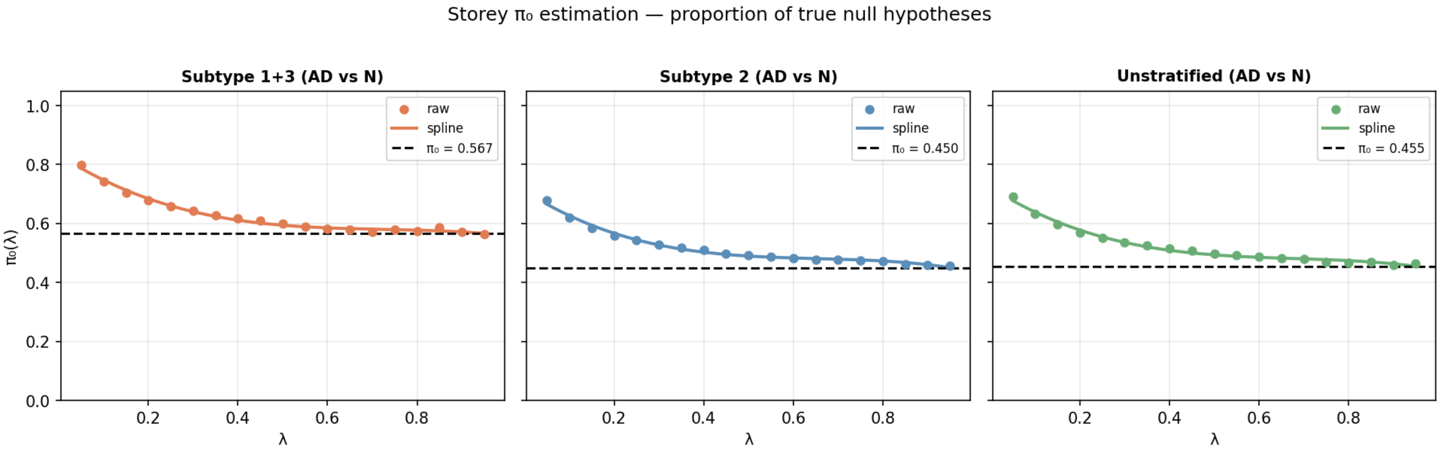

b

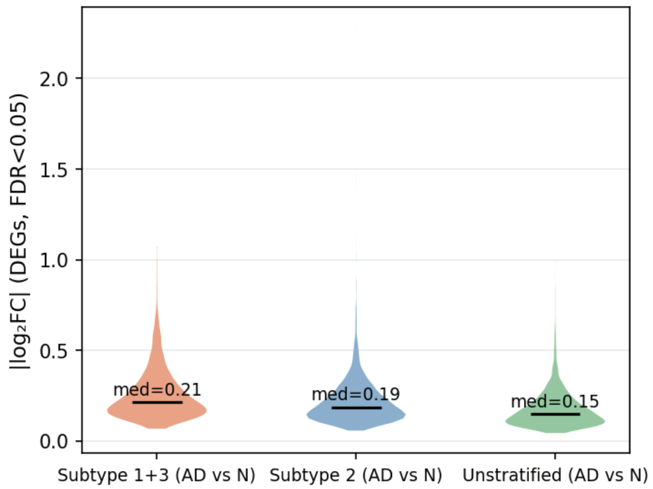

Figure S11.

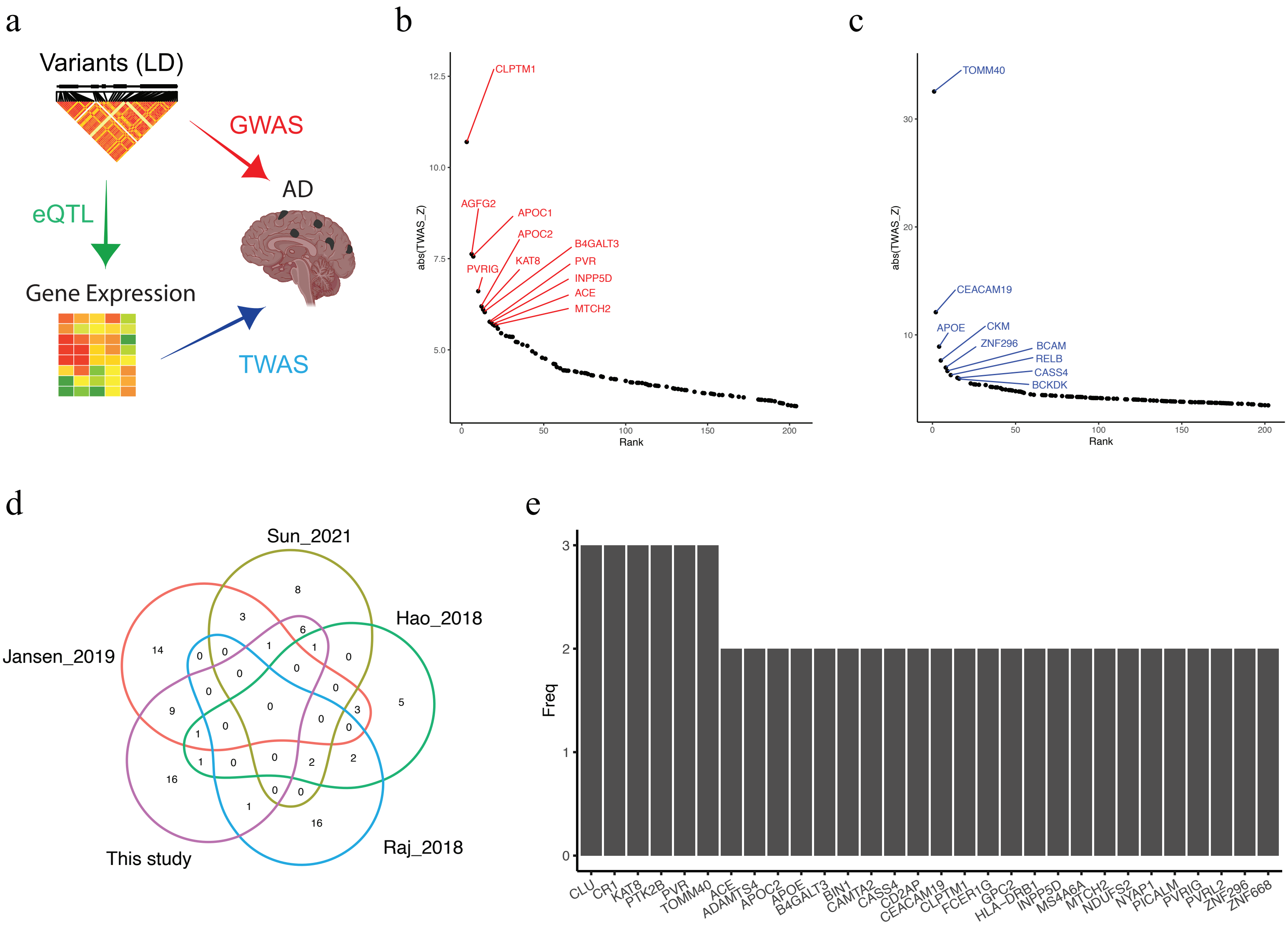
a
Variants (LD)
GWAS
AD
eQTL
Gene Expression
TWAS
b
CLPTM1
AGFG2
APOC1
APOC2
B4GALT3
KAT8
PVR
PVRIG
INPP5D
ACE
MTCH2
abs(TWAS_Z)
Rank
c
TOMM40
CEACAM19
APOE
CKM
ZNF296
BCAM
RELB
CASS4
BCKDK
abs(TWAS_Z)
Rank
d
Sun_2021
Hao_2018
Jansen_2019
Raj_2018
This study
e
Freq
CLU
CR1
KAT8
PTK2B
PVR
TOMM40
ACE
ADAMTS4
APOC2
APOE
B4GALT3
BIN1
CAMTA2
CASS4
CD2AP
CEACAM19
CLPTM1
FCER1G
GPC2
HLA-DRB1
INPP5D
MS4A6A
MTCH2
NDUFS2
NYAP1
PICALM
PVRIG
PVRL2
ZNF296
ZNF668

Figure S12

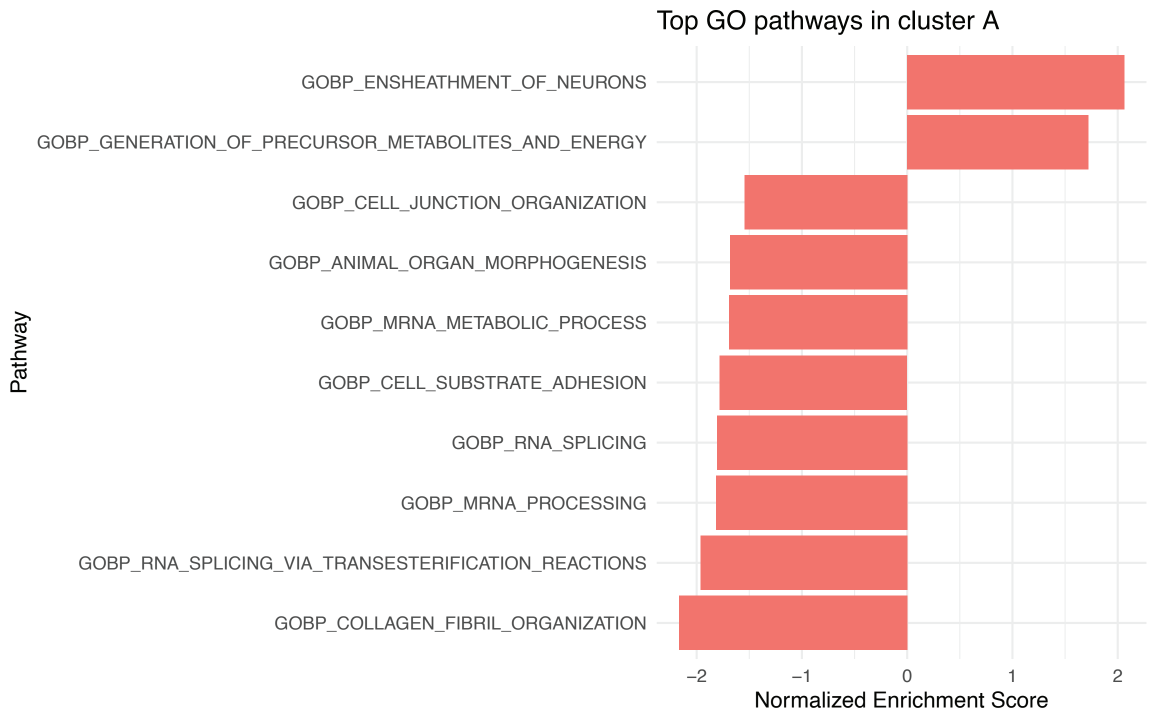
Top GO pathways in cluster A
GOBP_ENSHEATHMENT_OF_NEURONS
GOBP_GENERATION_OF_PRECURSOR_METABOLITES_AND_ENERGY
GOBP_CELL_JUNCTION_ORGANIZATION
GOBP_ANIMAL_ORGAN_MORPHOGENESIS
GOBP_MRNA_METABOLIC_PROCESS
GOBP_CELL_SUBSTRATE_ADHESION
GOBP_RNA_SPLICING
GOBP_MRNA_PROCESSING
GOBP_RNA_SPLICING_VIA_TRANSESTERIFICATION_REACTIONS
GOBP_COLLAGEN_FIBRIL_ORGANIZATION
Pathway
−2
−1
0
1
2
Normalized Enrichment Score
