# Supplementary material for: Latent Factor Modeling Reveals Unexpected Spatial Heterogeneity in Human Alzheimer’s Disease Brain Transcriptomes
Source: Comput Struct Biotechnol J. 2026 May 14;35(1):0108. doi: 10.34133/csbj.0108 (PMC13172580; doi:10.34133/csbj.0108)
Supplement: Supplementary 1 — Figs. S1 to S12 Tables S1 to S7 [file csbj.0108.f1.zip › Supplementary legends.docx]

**Supplemental information titles and legends**

**Figure S1. Sensitivity analysis of clustering stability across parameter choices.** Mean Adjusted Rand Index (ARI) is shown across ranks (k) for different numbers of features (topVal), regularization parameters (λ), and semi-NMF runs (nRun).

**Figure S2. GO and KEGG annotations of subgroup-specific signature genes.** NES: Normalized Enrichment Score.

**Figure S3. Associations between clinical variables and the latent factor**. **a**, Violin plots displaying the level of clinical and technical factors across the 3 subgroups. No significant differences were found among the subgroups except for neuroticism. **b**, the number of samples in each subgroup across the 9 batches of ROSMAP cohort.

**Figure S4. Evaluation of the random forest classifier**. **a**, The out of bag AUC curves across the 3 subgroups. **b**, the prediction scores in each subgroup in the ROSMAP cohort.

**Figure S5. Distribution of log2 fold changes of previously characterized layer markers in comparing each subgroup against the rest using ROSMAP RNA-seq samples.**

**Figure S6. Prediction scores of the 3 subgroups on each spatial spot of 12 spatial transcriptomics samples from a published study (Kristen et al.).** The prediction scores were z- score normalized.

**Figure S7. Variance of expression explained by clinical and technical variables and the latent factor using the MSBB cohorts (BM10, BM22, BM36, BM44).** SEX: gender batch: RNA-seq batch; diag: diagnosis of AD; RACE: ethnical groups.

**Figure S8. Overlap between the subgroup-enriched genes and the AD modules from Mostafavi’s study.** Subgroup 3 samples were merged into subgroup 1. X-axis is the percentage of module genes that overlapped with subgroup signature genes, y-axis shows the modules that significantly overlapped with either subgroup 1 or subgroup 2 signature genes. Dots with larger sizes represent higher significant levels with red color indicating adjusted p-value <= 0.05.

**Figure S9. Correlation between random forest subgroup 2 prediction scores and white matter marker scores**. **a**, boxplot showing the single sample GSEA (ssGSEA) scores of WM markers in each subgroup of the ROSMAP RNA-seq samples. **b**, the correlation between the ssGSEA scores and the subgroup 2 prediction scores. The WM markers were obtained from Maynard et al.

**Figure S10. Evaluation of subgroup-specific DEGs in AD versus normal in ROSMAP**. **a**, Storey π₀ estimates for the three differential expression analyses, calculated by fitting a natural cubic spline to the proportion of p-values exceeding each threshold λ. **b**, log2 fold changes of the significant genes (FDR < 0.05).

**Figure S11. Subgroup-specific expression patterns of putative AD-driver genes identified through TWAS**. **a**, Schematic representation of the transcriptome-wide association study (TWAS) that integrates both genome-wide association study and expression quantitative trait loci (eQTL). Summary statistics of 6 AD-GWAS, and eQTL models built on GTEx brain samples were used. **b**, Risk genes ranked by z-scores computed by using the FUSION package. Six AD GWAS summary results were used to calculate the z-scores, and the largest z-score of the 6 scores was used as the TWAS z-score for each gene. **c**, Protective genes ranked by z-scores. **d**, Intersections of TWAS significant genes identified from this study and 4 published studies. **e**. Frequency of each gene identified by TWAS. Genes reported by more than 1 study are used.

**Figure S12. Significantly up- and down-regulated GO pathways in proteome cluster A.**

**Table S1. Summary of sample sizes of RNA-seq datasets.**

**Table S2. Differentially genes in each cluster as compared to the rest.**

**Table S3. Definition of AD co-expression modules derived from MSBB bulk RNA-seq.**

**Table S4. Evaluation of DEG discovery under different settings.**

**Table S5. Differential metabolites in cluster A versus cluster B.**

**Table S6. Published metabolite markers for WM and GM.**

**Table S7. Differential proteins in cluster A versus cluster B.**
